# Ultra-low-dose CBCT scan: rational map for ear surgery

**DOI:** 10.1007/s00405-022-07592-4

**Published:** 2022-09-16

**Authors:** Pekka Tamminen, Jorma Järnstedt, Antti Lehtinen, Jura Numminen, Lauri Lehtimäki, Markus Rautiainen, Ilkka Kivekäs

**Affiliations:** 1 Department of Otorhinolaryngology–Head and Neck Surgery, Tampere University Hospital, Elämänaukio 2, 33520 Tampere, Finland; 2 Department of Otorhinolaryngology, Satasairaala, Sairaalantie 3, 28500 Pori, Finland; 3 Department of Internal Medicine, Tampere University Hospital, Elämänaukio 2, 33520 Tampere, Finland; 4 Medical Imaging Centre, Department of Radiology, Tampere University Hospital, Teiskontie 35, 33520 Tampere, Finland; 5 Allergy Centre, Tampere University Hospital, Elämänaukio 2, 33520 Tampere, Finland; 6 Faculty of Medicine and Health Technology, Tampere University, Arvo Ylpön Katu 34, 33520 Tampere, Finland

**Keywords:** Cone beam computed tomography, Ultra-low-dose protocol, Temporal bone, Image quality, Ear surgery

## Abstract

**Purpose:**

This study will evaluate the clinical quality and usability of peripheral image data from the temporal bone area obtained using a sinonasal ultra-low-dose (ULD) cone-beam computed tomography (CBCT) scan and compare them to those obtained using a high-resolution (HR) CBCT.

**Methods:**

The population consisted of 66 anatomical sites (ears of 33 subjects) imaged using two modalities: an HR CBCT (Scanora 3Dx scanner; Soredex, Tuusula, Finland) and a ULD CBCT (Promax 3D Mid scanner; Plandent, Helsinki, Finland). The image quality (IQ) for every anatomical site in each image was rated using a Likert scale from 0 to 5.

**Results:**

The quality of ULD CBCT scans was clinically sufficient in over 95% of the assessed images of the sigmoid sinus, jugular bulb, epitympanum and mastoid antrum as well as external acoustic meatus (all *p* > 0.05 compared to HR CBCT). The IQ was clinically sufficient in 75–94% of the assessed images of the scutum, mastoid segment of the facial nerve, cochlea and semicircular canals (all *p* < 0.05 compared to HR CBCT). The overall IQ of the HR CBCT scans was good or excellent.

**Conclusion:**

CBCT imaging and the data at image margins are underutilized. CBCT can produce excellent structural resolution with conventional imaging parameters, even with off-focus images. Using ultra-low doses of radiation, the produced IQ is clinically sufficient. We encourage ear surgeons to check the patients’ imaging history and to consider the use of imaging modalities that involve lower radiation doses especially when conducting repetitive investigations and with children.

## Introduction

The basic aim of otologic imaging is twofold: firstly, to determine the disease or pathology, for example to evaluate the extent of chronic inflammation or cholesteatoma, and secondly, to explore an individual’s anatomy before surgery.

In Finland, radiation-based imaging of the temporal bone is done roughly 1050 times a year (pre-COVID-19 5-year mean, 2015–2019). Cone-beam computed tomography (CBCT) accounts for only 65 (6%) of these images [1]. An annual mean (2015–2019) of 1100 surgical procedures were conducted on ossicles, inner ears, mastoids or temporal bones in Finland [1].

CBCT was first introduced in the late 1990s, and it has been improved significantly since then. An image is taken with a two-dimensional detector that rotates around the object 180°–360° [2]. Imaging is usually done with the patient sitting upright with stabilizing head support. Although this method is convenient, motion artefacts related to the patient’s position are one of the weaknesses of CBCT [2–4]. The image data consist of small isotropic 3D units, or voxels, that represents the degree of X-ray absorption at each location [2]. The size of the voxel determine the image resolution, and the resulting image can be rendered and re-rendered freely. In the field of otorhinolaryngology, CBCT has multiple applications; the most common is imaging of the sinonasal cavities.

As early as 15 years ago, Peltonen et al. [5] demonstrated the substantial potential of CBCT for imaging the middle ear; however, multi-slice computed tomography (MSCT) is still the standard method. The most recent articles rank CBCT as equal or superior to MSCT in terms of image quality (IQ) and resolution [6–8]. However, these studies compared images of cadaver heads and are thus free of real-life motion artifacts. In-vivo comparisons of the modalities find that the images are equivalent, although MSCT is better for imaging inner ear structures and CBCT produces better images of the ossicles [9]. Another study state that MSCT is equal or better; CBCT producing adequate or very good images for 75–85% of the evaluated structures [10].

Most existing studies comparing these imaging types focus on IQ, resolution or contrast-to-noise-ratio. However, is it always necessary to use larger doses of radiation to obtain clearer images? The use of high-resolution (HR) CBCT can reduce radiation doses by 70% compared to MSCT [6]. Optimizing radiation exposure is extremely important when imaging children because of the increased risk of cancer due to lifetime radiation exposure [11].

The guiding principle of radiation safety indicates that we should aim for radiation doses that are as low as diagnostically achievable (ALADA) [11]. Therefore, CBCT, with its developing increased computational power and patients image archive, are worth exploring.

Our aims were, firstly, to evaluate the clinical quality and usability of peripheral image data for the temporal bone area obtained using a sinonasal ultra-low-dose (ULD) CBCT scan on clinical perspective and, secondly, to identify the strengths and weaknesses of this imaging modality.

## Methods

The included subjects had chronic or recurrent rhinosinusitis and were referred to the Department of Otorhinolaryngology of (Tampere) university hospital. HR CBCT and ULD CBCT images were taken four to six weeks apart in accordance with the follow-up protocol of our ongoing study.

The HR CBCT scans were obtained using a Scanora 3Dx scanner, (Soredex, Tuusula, Finland) with the following specifications: field of view (FOV): 140 × 165 mm, 90 kV, 8 mA, scanning time: 4 s, voxel size: 0,2 (high resolution), slice thickness: 1 mm, spacing: 1 mm, zoom: 1 and dose area product (DAP): 1725.85 mGycm2.

The ULD CBCT scans were obtained using a Promax 3D Mid scanner (Plandent, Helsinki, Finland) with the following specifications: FOV: 160 × 170, 120 kV, 2 mA, two scans stitched together by Romexis software, scanning time: 4.486 s and 4.522 s, voxel size: 0.6 (ultra-low dose), slice thickness: 1.4 mm, spacing: 1.4 mm, zoom: 0.8 and DAP: 2 × 115 (= 230) mGycm2.

Standard axial, coronal and sagittal reconstruction were done using the Philips IntelliSpace Portal v10.1.5.51377 workstation (Philips Medical Systems, Netherlands). No enhancements were used. To maximize overall IQ, different reconstructions were chosen based on the radiological department’s previous experience with ULD imaging. All the original and reconstructed slices were stored in the picture archiving and communication systems (PACS) of the radiological department.

Three raters (JJ, IK and PT: a radiologist (23), an ear surgeon/neuro-otologist (11) and a general otorhinolaryngologist (6); years of clinical experience as a specialist are indicated in brackets) assessed the images separately. A Sectra IDS7 PACS workstation and a Barco MXRT 4700 Dicom monitor with a resolution of 2560 × 1600 (60 Hz) was used. All scans were recorded with a random, unique number from 1 to 1000 and rearranged in ascending order. The image metadata were hidden (including name and modality), and fixed planes (axial, coronal and sagittal) were used. The sharpness of the images could be adjusted freely.

The IQ for every anatomical site (Table 2) in each image was rated using a Likert scale (Table 1) from 0 to 5: 0 = cannot be assessed; 1 = poor IQ; 2 = reduced IQ; 3 = acceptable IQ; 4 = good IQ; 5 = excellent IQ. Ratings from 0 to 2 were considered as insufficient; ratings from 3 to 5 were considered sufficient for anatomical guidance and for pre-operative decisions.

**Table 1:** Likert scale from 0 to 5 used to assess the image quality of HR CBCT and ULD CBCT images

		Structure	Quality
0	Cannot be assessed	No identifiable structure or other reason	
1	Poor image quality	Some anatomic resemblance	Major image noise or artefacts
2	Reduced image quality	Poorly defined anatomic details	High image noise or artefacts
3*	Acceptable image quality	Limitations in anatomic detail	Increased image noise or artefacts
4	Good image quality	Clear anatomic details	Minor image noise or artefacts
5	Excellent image quality	Distinct anatomic details	No or minimal image noise or artefacts

*If an overall understanding of the structures anatomy was received, the grade was three or more, if not, grade two, one or zero was given. Exemplary questions to guide the rating: Can the course of the structure be followed? Can thin bony walls/structures be identified?

### Statistical analysis

The statistical analysis was conducted using SPSS Statistics 27.0.1.0 (International Business Machines Corporation (IBM), Armonk, USA). Kendall’s coefficient of concordance (W) was used to assess inter-rater agreement for the three raters. Fisher’s exact test (two-tailed) or Chi-squared (Pearson) were used for cross-classifiable results.

### Ethical statement

The study was approved by the (Tampere university hospital) ethical committee (R17011 and registered at clinicaltrials.gov; NCT04171167) and all patients provided written informed consent. Repetitive imaging was conducted ethically; the additional radiation burden was equivalent to a few days of background radiation.

## Results

The study population consisted of 66 anatomical sites (both ears from 33 subjects) imaged using two modalities: HR CBCT and ULD CBCT.

The ULD CBCT images of the sigmoid sinus, jugular bulb, epitympanum and mastoid antrum as well as external acustic meatus were of clinically sufficient quality. For these structures, over 95% of the scans received a rating of 3–5; these ratings did not differ significantly to those of the HR CBCT images (Table 2.). The images of the scutum, mastoid segment of the facial nerve, cochlea and semicircular canals were rated as clinically sufficient 75–94% of the time. The incudomalleolar joint and tympanic segment of the facial nerve were the most challenging structures for ULD CBCT imaging modality; nonetheless, 42–43% of these images were rated as sufficient.

**Table 2:** Image quality comparison and inter-rater agreement for high resolution CBCT and ultra-low dose CBCT images

	CBCT modality	Total*	Image quality										
			Likert scale						Inter-rater agreement		Insufficient (0–2)	Sufficient (3–5)	*p* value
			0	1	2	3	4	5	Kendall’s W	*p* value	%	%	
Sigmoid sinus	ULD	144	4	0	2	24	113	1	0.420	0.106	4.2	95.8	0.062^1^
	HR	150	1	0	0	3	33	113	0.396	0.172	0.7	99.3	
Jugular bulb	ULD	195	3	0	3	31	155	3	0.383	0.192	3.1	96.9	0.284^1^
	HR	189	2	0	0	3	50	134	0.447	**0.038**	1.1	98.9	
Incudomalleolar joint	ULD	186	2	21	84	59	20	0	0.405	0.120	57.5	42.5	** < 0.001**^**2**^
	HR	192	0	0	5	44	95	48	0.470	**0.018**	2.6	97.4	
Semicircular canals	ULD	192	4	5	32	31	112	8	0.321	0.561	21.4	78.6	** < 0.001**^**1**^
	HR	186	2	0	2	10	50	122	0.389	0.173	2.2	97.8	
Cochlea	ULD	198	0	0	27	72	96	3	0.505	**0.005**	13.6	86.4	** < 0.001**^**1**^
	HR	192	0	0	0	18	74	100	0.354	0.354	0.0	100.0	
Epitympanum and mastoid antrum	ULD	177	0	0	3	44	124	6	0.524	**0.004**	1.7	98.3	0.248^1^
	HR	177	0	0	0	0	57	120	0.372	0.253	0.0	100.0	
Tympanic segment of facial nerve	ULD	195	4	29	80	49	33	0	0.344	0.404	57.9	42.1	** < 0.001**^**1**^
	HR	195	2	0	2	22	116	53	0.478	**0.013**	2.1	97.9	
Mastoid segment of facial nerve	ULD	186	3	15	28	46	92	2	0.406	0.118	24.7	75.3	** < 0.001**^**1**^
	HR	180	0	0	1	6	45	128	0.338	0.446	0.6	99.4	
Scutum	ULD	189	1	1	10	62	108	7	0.457	**0.028**	6.3	93.7	** < 0.001**^**1**^
	HR	192	0	0	0	2	54	136	0.487	**0.010**	0.0	100.0	
External acoustic meatus	ULD	189	4	0	0	53	131	1	0.554	** < 0.001**	2.1	97.9	1^1^
	HR	186	4	0	0	0	81	101	0.445	**0.041**	2.2	97.8	

*ULD* ultra-low-dose conebeam computed tomography, *HR* high-resolution conebeam computed tomography

*Only image pairs in which structures are visible, both sides are evaluated independently

Statistical analysis: ^1^Fishers exact test (two-tailed)

^2^Chi-square (Pearson); The level of significance was set at *p* < 0.05 (indicated with bold font)

Overall, the quality of the HR CBCT images was good or excellent. Almost all the images (97–100%) were rated clinically sufficient. Occasionally, lower ratings were given to structures such as the incudomalleolar joint, semicircular canals, and tympanic and mastoid segments of the facial nerve.

**Fig. 1:**
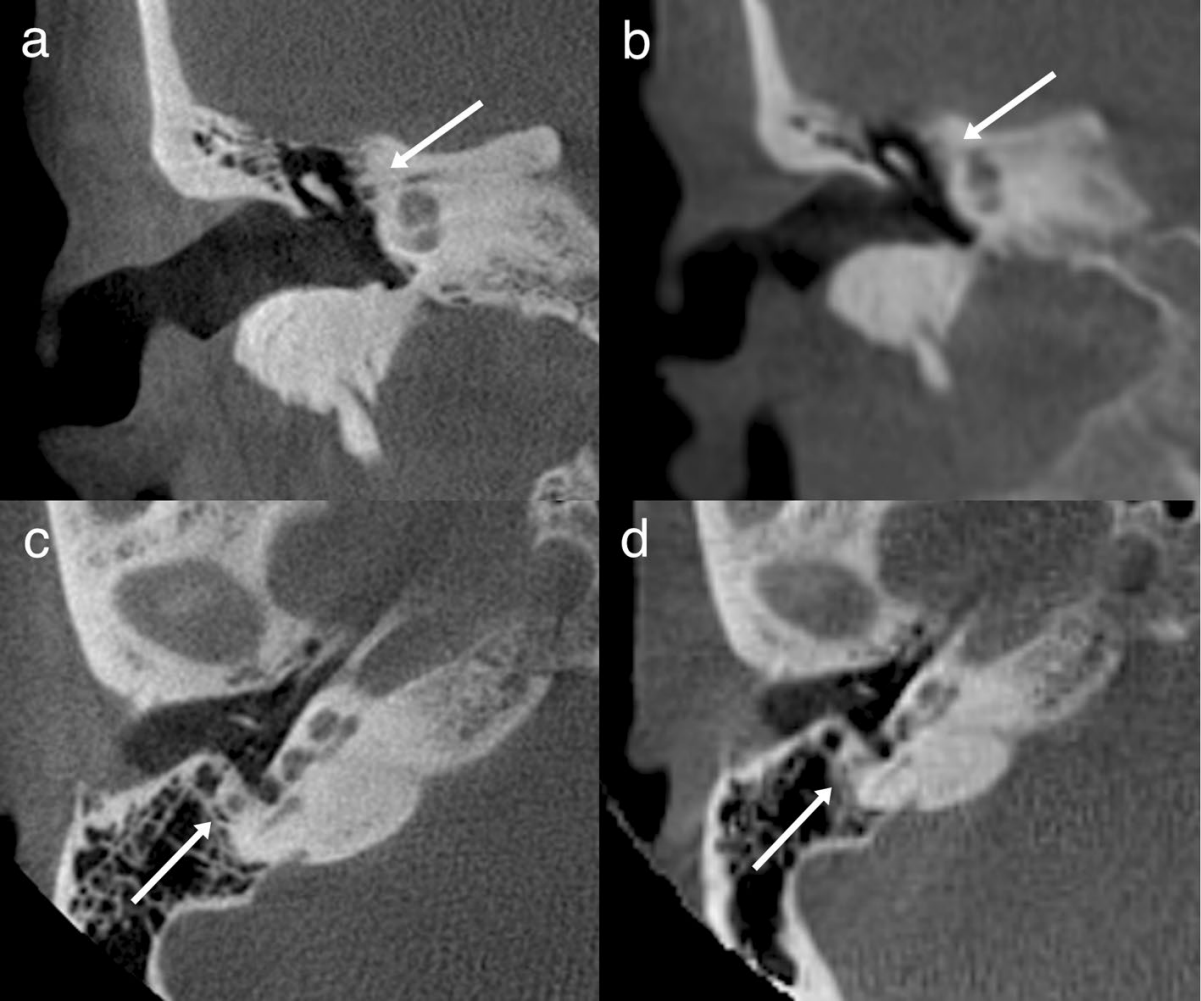
Two example images from the same patient: panels **a** and **c** were taken with HR CBCT and panels **b** and **d** with ULD CBCT. Both scans were rated as high quality. Panels **a** and **b** are in the coronal plane, and panels **c** and **d** are in the axial plane. The course of the facial nerve is visible

**Fig. 2:**
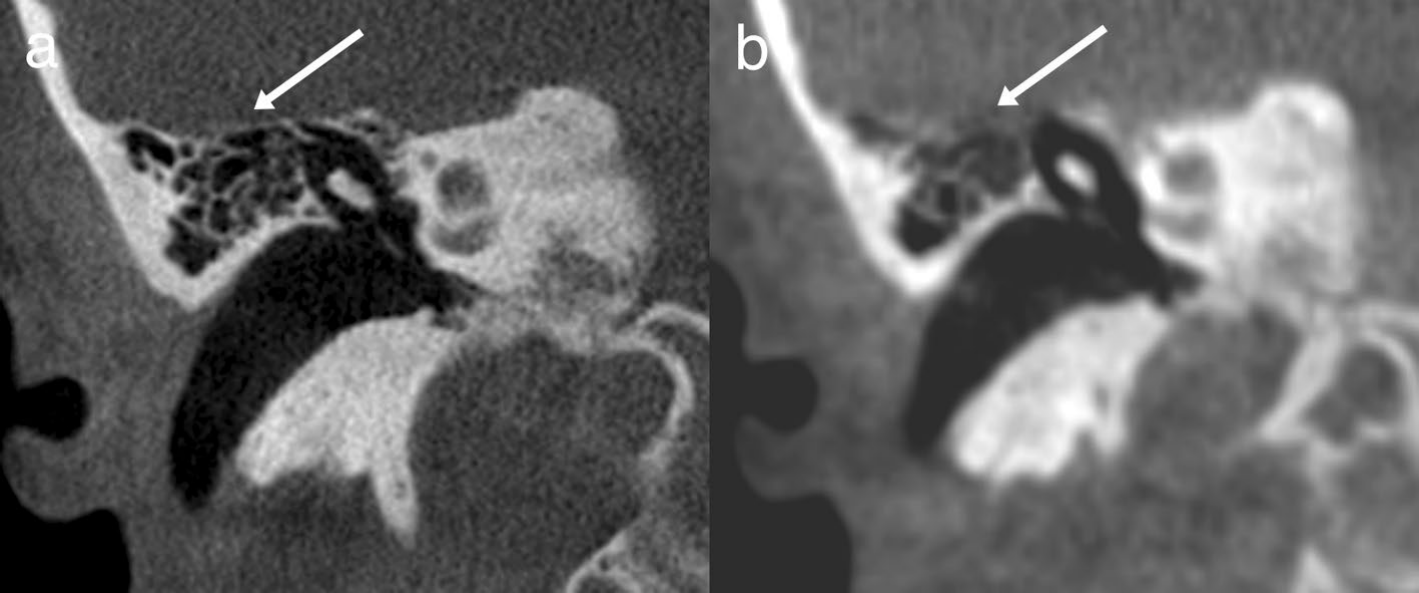
Example images in the coronal plane from a single patient, one taken with HR CBCT (**a**) and one with ULD CBCT (**b**). Fine bone structures, such as tegmen tympani, are not always identifiable in the ULD CBCT image

**Fig. 3:**
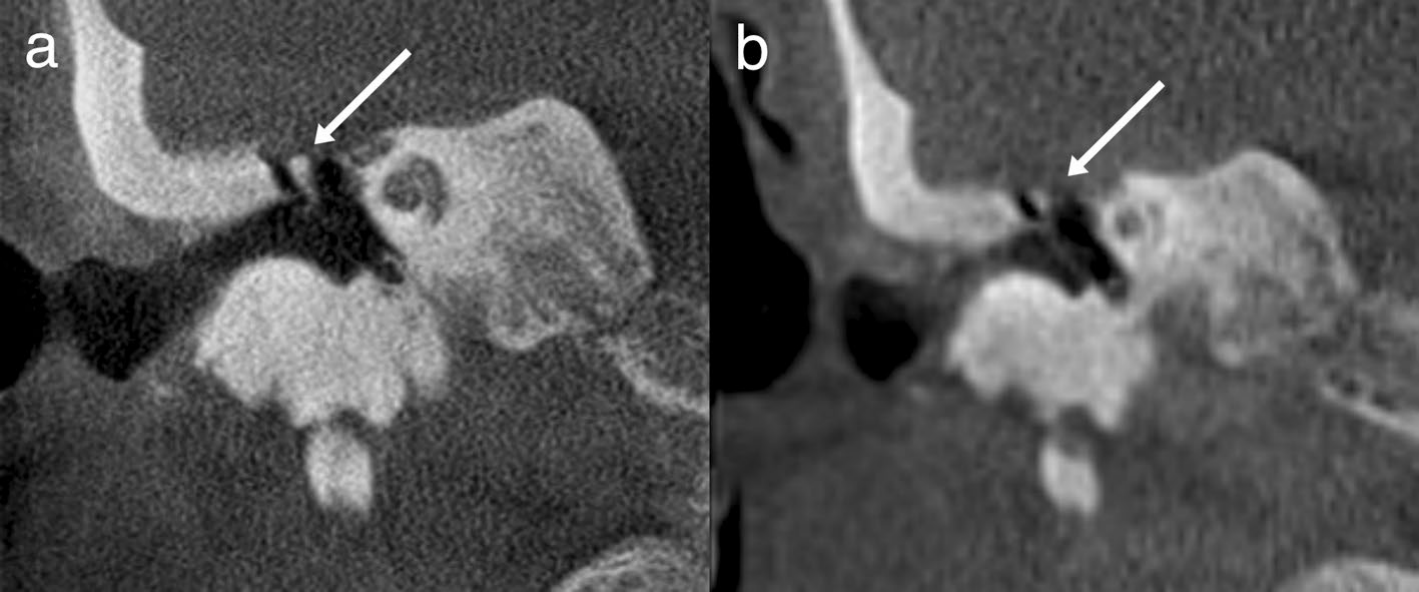
Example images in the coronal plane from a single patient, one taken with HR CBCT (**a**) and one with ULD CBCT (**b**). The meningoencephalocele protrudes through the tegmen tympani and is in contract with the incus and the malleus

**Fig. 4:**
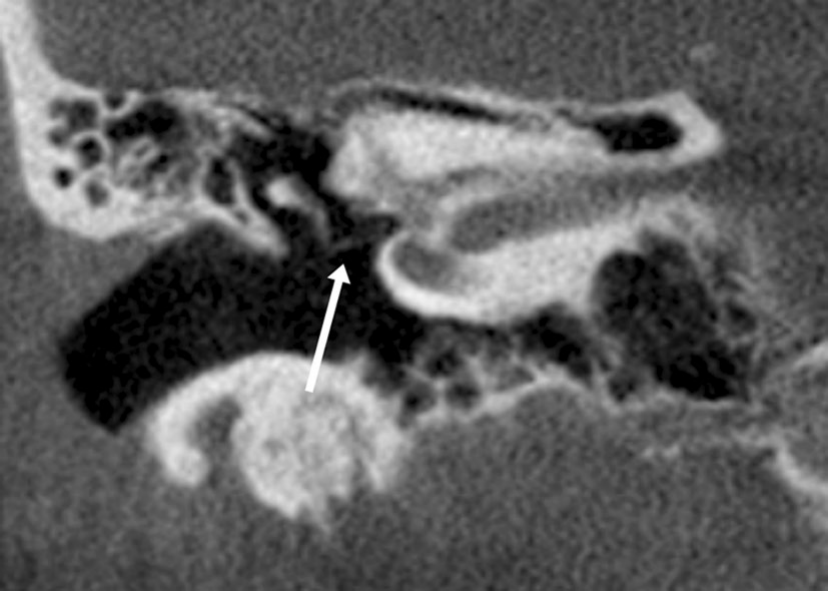
Example images in the coronal plane taken with HR CBCT demonstrate the capabilities and limitations of off-focus images for imaging the fine ossicle structures of the incus and the stapes

**Fig. 5:**
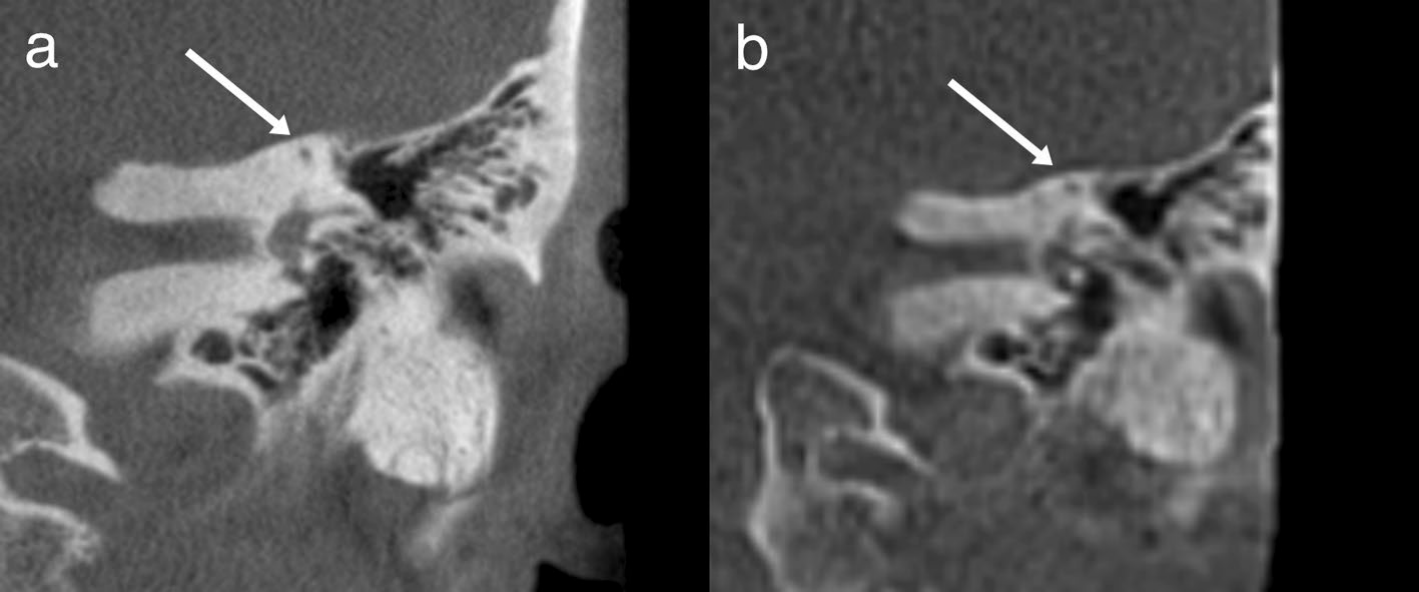
Example images in the coronal plane taken from a single patient, one with HR CBCT (**a**) and one with ULD CBCT (**b**). The bony coverage of the superior semi-circular canal is visible

The ULD CBCT images were noticeably softer than the HR CBCT images (Fig. 1). The stitching area was directly on the evaluated structures, which in some case negatively impacted the IQ. The largest difference in IQ between the two imaging modalities could be observed in images of fine bony structures. Air-filled cavities provided a clear contrast for all structures, but the integrity of the bony roof of the mastoid cavity or the tegmen tympani, for example, was not always evident (Fig. 2). However, we did diagnose one patient with meningoencephalocele through the tegmen tympani (Fig. 3). The pathology was clearly visible in the HR CBCT scan and identifiable in the ULD CBCT. Similar IQ differences between HR CBCT and ULD CBCT were associated with the facial nerve, to a lesser extent during its course through the mastoid bone and more so in the tympanic segment. In scans with sufficient IQ, it was not difficult to identify the course (Fig. 1). The potential thin bony coverage of the nerve in the tympanic segment was noticeably harder to identify in the ULD CBCT image, if it could be observed at all. In some HR CBCT images, however, even the stapes was visible (Fig. 4). The integrity of the semicircular canal bone could be assessed in images taken with both modalities, but it was only possible in ULD CBCT images if the patient’s anatomy was favourable (Fig. 5).

Inter-rater agreement was highest and in the scutum and the external acoustic meatus (Table 1).

## Discussion

To our knowledge, the present study is the first to compare and evaluate sequential ULD temporal bone CBCT images to conventional HR CBCT images in a clinical cohort. Repetitive imaging without changes in pathology or anatomy is essential for comparing imaging modalities. There are only few clinical studies comparing the use of MSCT and CBCT to temporal bone imaging: Redfors et al. [10] were the first to evaluated the anatomical structures of the temporal bones of 20 post-stapedectomy patients (otosclerosis), and Pein et al. [9] compared images of 38 patients without pathology, but most of the compared images were from different patients. Some additional studies comparing imaging modalities have a narrow scope, such as imaging post-operative implant position or pre-operative imaging of otosclerosis.

The present study demonstrates that the IQ of ULD CBCT scans and especially that of HR CBCT scans is sufficient for several procedures in the field of ear surgery, even if off-focus scans are used. Both methods provide an anatomical map of relatively constant structures of the temporal bone, such as the external acoustic meatus, sigmoid sinus, jugular bulb, cochlea, semicircular canals and the mastoid segment of the facial nerve. It might also be possible to identify focal pathologies from ULD CBCT scans of the epitympanum, mastoid antrum or scutum.

High-resolution computed tomography (HRCT) is the gold standard for imaging the temporal bone. This method provides crisp, clear images of the bony structure with radiation doses ranging from 1.6 mSv for adults to 7.1 mSv for new-borns, an amount equivalent to 6–24 months of background radiation [8, 12]. CBCT can produce images that are as good or even better [5–7, 9]. However, the hypernym CBCT includes a variety of hardware, software and setting combinations, our combinations in this article are named as HR CBCT and ULD CBCT. Since the present study uses a retrospective design, we did not measure the effective dose of radiation. Furthermore, no previous studies have reported the effective radiation doses for these scanners in this imaging area. The overall mean effective doses have been published: 0.119 mSv for paranasal CBCT [13] and 0.6 mSv for paranasal MSCT [14], equivalent to two to nine weeks of background radiation. The effective dose for facial scans with the Scanora 3Dx (HR CBCT device) is reported to be 0.104 mSv (unpublished data from the manufacturer; measured in the University Hospital of Oulu, Finland, in 2013, according to the dosimetric principles of the Finnish Radiation and Nuclear Safety Authority [STUK]). For facial imaging with the Promax 3D Mid ULD (ULD CBCT device), the dose is 0.018 mSv (FOV: 200 × 170 mm) (published in a poster; ID 0920, 2015 IADR/AADR/CADR General Session, Boston Massachusetts), which equals two days of background radiation. The facial area includes radiation-sensitive organs, such as the thyroid and the submandibular glands, which were not in our FOV; but we had two 160 × 170 mm stacks instead of one 200 × 170 mm. The ULD CBCT uses six or seven times less radiation than the HR CBCT, as seen in the lower mSv and DAP values. If the ULD CBCT images were obtained in one scan focused on the ears, this reduction would double.

For our CBCT scans, we used a sinonasal focus as a reference. Although our HR CBCT is not compared to HRCT, the excellent IQ of HR CBCT is demonstrated in the samples and results. The IQ of CBCT images could be further enhanced if the patient were supine during scanning (this would reduce motion artefact) and by adjusting the primary focal area on the ear. In optimal circumstances (in vitro), superb resolution of fine structures (stapes, incudostapedial joint, tendon of the tensor tympani and the stapes footplate, to name a few) is possible with CBCT (tube voltage: 88 kV; tube current: 11 mA; voxel size: 0.1 mm; FOV: 60 × 60 mm) [5, 7]. We knew from experience that ULD CBCT with our specifications is not likely to produce clinically sufficient images of these above mentioned structures, hence majority of them were not even included in our list of structures. The results support our assumption.

It is preferable to obtain the best image possible, particularly when confirmation of a structure’s normality can rule out the need for explorative surgery or surgical intervention. For example, even when detailed images are available, tympanotomy is often performed to verify, for instance, the status and mobility of the ossicles or the footplate and, if needed, to remove the pathology and reconstruct the chain’s integrity.

Cochlear implantations are a good example of a surgical procedure conducted on ‘healthy’ ears; there is no consensus regarding the best pre-operative imaging modality [12]. Of children and adults who receive cochlear implantations, 85% undergo magnetic resonance imaging (MRI), 95% of children and 90% of adults undergo CTs and around 80% receive both [15]. We consider the MRI a baseline measurement for both children and adults as it provides a comprehensive view of the neural and inner ear structures [16–19]. Some authors recommend proceeding to surgery without imaging if there is no clinical or audiometric concerns for otosclerosis or middle ear and/or mastoid disease [15, 20]. We suggest that an ultra-low-radiation dose method of imaging, such as a ULD CBCT, could be used as the first approach to CT in children and adults with suspected healthy ears. This type of imaging can be used to evaluate the anatomical relationships of the critical structures, such as the sinus sigmoideus, facial nerve and jugular bulbus. With these said, we are still waiting our CBCT device that allows supine position, and thus enables even young children’s imaging. If obscure structures are found and the MRI does not provide enough information, reveals cochlear anomaly or some unexpected pathology, more comprehensive CT imaging (HR CBCT or MSCT) is justified.

Furthermore, there is frequently a vast reservoir of unused information in CT and CBCT images that have already been taken. In the field of otorhinolaryngology, these images focus primarily on the nasal cavity and paranasal structures, but all corners of the FOV should be utilized. The reviewer, radiologists or clinician is responsible for analysing the entire imaged area [21]. Our results show that recent imaging history might be useful. If a new image is needed for post-operative monitoring or for other non-complicated reasons, it could also be sufficient to take this image using a considerably lower radiation dose.

One strength of our study is that we used comparable images of real subjects. The images were rated in a random order, and the raters had no information about the patients or the imaging modalities. In addition, the images were viewed in same planes and with the same desktop options that clinicians normally use. However, no technical evaluation or comparison of the images was conducted, as we considered such an evaluation irrelevant to our objective. Another weaknesses of our study is that all the imaged ears were healthy. As a result, the findings cannot be generalized to ears with considerable pathology, and no surgery was planned based on our images.

Inter-rater argeement is moderate at its best regardless of the modality. Nevertheless, our findings reflect real-world circumstances, as the raters included a radiologist, an ear surgeon/neuro-otologist and a general otorhinolaryngologist. In order to remain strictly critical, every individual rating from 0 to 2 impaired the insufficient-sufficient ratio.

The ULD images in our data are combined from two stacks with an overlap of about two centimeters; this overlap is the stitching area. Its position varies according to each patient’s individual anatomy, but it usually includes the structures of interest. In an ideal situation, excess computing and geometric distortion [2] may be avoided by adjusting the FOV to include only the structures that are needed and setting the focus accordingly.

To conclude, CBCT imaging and the data at image margins are underutilized. CBCT can produce excellent structural resolution with conventional imaging parameters, even with off-focus images, and it can produce images of clinically sufficient image quality with an ultra-low dose of radiation. We encourage ear surgeons to check patients’ imaging history and to consider the use of imaging modalities that involve lower radiation doses especially for repetitive investigations and with children.
